# Development and Internal Validation of a Predictive Model for Operative Management in Blunt Abdominal Trauma Using Admission Physiological and Biochemical Parameters

**DOI:** 10.3390/jcm14238379

**Published:** 2025-11-26

**Authors:** Raúl Sampayo-Candia, Carlos A. Guzmán-Martín, Miguel A. Vázquez-Toledo, Fausto Sánchez-Muñoz, Alejandro Berruecos-Romero, Daniel Juárez-Villa, Ana Karen García-Hernández, Adriana Hernández-García, Belén Marisol Chávez-Alba, Iván Zepeda-Quiroz, Demian Trueba-Lozano

**Affiliations:** 1 Departamento de Cirugía de Trauma, Hospital de Traumatología y Ortopedia Dr. y Gral. Rafael Moreno Valle, Servicios Públicos de Salud del Instituto Mexicano del Seguro Social Para el Bienestar (IMSS-BIENESTAR), Puebla 72573, Mexico; md.sampayo@icloud.com (R.S.-C.); alejandro.berruecos1c@gmail.com (A.B.-R.); akareengh@gmail.com (A.K.G.-H.); adri.hega@gmail.com (A.H.-G.); ilm_belen@hotmail.com (B.M.C.-A.); 2 Doctorado en Ciencias Biológicas y de la Salud, Universidad Autónoma Metropolitana, Ciudad de México 14387, Mexico; gmcarlos93@gmail.com; 3 Departamento de Fisiología, Instituto Nacional de Cardiología Ignacio Chávez, Ciudad de México 14080, Mexico; fausto22@yahoo.com; 4 Posgrado en Ciencias en Inmunología, Escuela Nacional de Ciencias Biológicas, Instituto Politécnico Nacional (ENCB-IPN), Ciudad de México 11340, Mexico; miguelpradyu1401@gmail.com; 5 Departamento de Medicina Interna, Hospital General de Zona No. 18, Instituto Mexicano del Seguro Social, Playa del Carmen 77710, Mexico; daniel_00_5@hotmail.com; 6 Departamento de Medicina Interna, Hospital Ángeles Puebla, Puebla 72190, Mexico

**Keywords:** blunt abdominal trauma, operative management, lactate, FAST, triage, logistic regression, prediction model, bootstrap validation

## Abstract

**Background:** Early identification of patients requiring operative management (OM) after blunt abdominal trauma is critical, yet initial physiological signs may be nonspecific. We sought to develop and internally validate an admission-based prediction model for early emergency department (ED) triage, prior to computed tomography (CT), using routinely available physiological and biochemical parameters. **Methods:** We conducted a retrospective observational study including adult patients with blunt abdominal trauma who underwent FAST and lactate testing at admission. OM was defined as any abdominal surgical intervention within 24 h to control hemorrhage or repair injury. A multivariable logistic regression model incorporating lactate, heart rate, leukocyte count, and FAST positivity was developed using complete-case data. Lactate diagnostic accuracy was assessed using ROC analysis. Internal validation was performed with 1000 bootstrap resamples. **Results:** In 81 patients with lactate results, lactate showed good discrimination for OM (AUC 0.815). At ≥3.5 mmol/L, sensitivity was 0.737 (95% CI 0.569–0.866), specificity 0.744 (0.588–0.865), LR^+^ 2.88, and LR^−^ 0.35. The final logistic model demonstrated an apparent AUC of 0.904 and an optimism-corrected AUC of 0.882. The full model equation and coefficients are provided for reproducibility. **Conclusions:** Admission lactate, combined with FAST and physiologic measurements, provides useful early-triage information before CT and warrants external validation in larger cohorts.

## 1. Introduction

Trauma is a multisystemic pathology that causes an increased and abnormal physiological demand by eliminating access to normal metabolic substrates [1]. In Mexico, injuries are a major cause of morbidity and mortality: in 2024, accidents and assaults reached 4th and 6th places as causes of death, according to the National Institute of Statistics and Geography (INEGI), with greater frequency in people in economically active years (15–34 years) and a mortality up to 50% [2,3].

Abdominal injuries are the third most common cause of organ damage (25–30%) and the most common traumatic cause of death (13–51%) [4,5,6,7,8]. In blunt trauma, damage occurs as a result of forces that exceed the strength of tissues; 70–90% due to high-energy mechanisms involving traffic incidents, 15% direct trauma to the abdomen, and 6–9% falls from a height [9,10,11,12]. The involvement of solid organs is common in blunt abdominal trauma (spleen 33–46%, liver 15–41.7%, and kidney 1–16.4%) and is associated with an estimated mortality of 10% (for spleen, liver, and kidney injuries), mainly due to bleeding [12,13,14,15,16,17].

Previously, it was considered that operative management (OM) had to be initiated early to reduce the morbidity and mortality of these injuries. Currently, international clinical practice supports the view that hemodynamically stable patients with blunt abdominal trauma—who can undergo continuous clinical monitoring and who do not present signs suggestive of associated injuries—are suitable candidates for non-operative management (NOM). This approach requires close follow-up through clinical evaluation, biochemical parameters, and complementary imaging studies, such as computed tomography (CT) [18,19,20,21,22,23].

Each patient’s condition (multiple injuries, recent use of alcohol or other drugs, etc.) influences his or her response to trauma and hemorrhage and cause a wide spectrum of clinical manifestations that make assessment and decision-making regarding the best therapeutic option difficult [24,25]. Although some studies claim that physical examinations have a 55–65% sensitivity to diagnosing injuries in blunt abdominal trauma, these numbers imply that the absence of physical findings does not rule out either injuries or the need for OM; therefore, additional assessment and diagnostic testing are required [26,27,28,29,30]. Laboratory tests in patients with severe abdominal injuries often reveal signs of massive blood loss, such as acidosis and coagulopathy, from the time of admission: lactate (>4 mmol/L), pH (<7.25), and base excess (BE, ≥−10) have demonstrated high prognostic value for mortality; for this reason, they must be monitored during treatment [31,32]. Furthermore, Yanar et al. observed that serum lactate levels on admission, the magnitude of solid organ injury (according to American Association for the Surgery of Trauma [AAST] injury scoring scale), the need for transfusion, crystalloid resuscitation, and drop in hematocrit within the first hour after admission are useful parameters to determine NOM failure [33,34,35,36].

Despite growing interest in serum lactate as a risk stratification tool, there is still limited consensus regarding its optimal cutoff point for guiding OM decisions in blunt abdominal trauma. Therefore, we aimed to develop and internally validate an admission-based multivariable model to estimate the probability of requiring operative management in blunt abdominal trauma. Importantly, this model is intended for early emergency department triage before CT, using variables available within minutes of arrival, and is not designed to replace CT-based decision-making or surgical judgment.

## 2. Materials and Methods

We conducted a retrospective observational study of adult patients with blunt abdominal trauma admitted to the “Doctor and General Rafael Moreno Valle” Traumatology and Orthopedics Hospital (Puebla, Mexico) between October 2022 and October 2024, approved by the institutional Ethics Committee (code: S2022-124-01, approval date: 7 October 2024). The model was designed for early emergency department (ED) triage before CT, using only variables available within minutes of arrival.

Patients ≥ 18 years old who underwent FAST and admission lactate testing were included; those requiring operative management (OM), defined as any abdominal surgical intervention performed within the first 24 h to control hemorrhage or repair intra-abdominal injury, were considered cases, and those successfully managed non-operatively (NOM) served as controls. All OM procedures were open laparotomies, as neither laparoscopy nor endovascular therapy is available at our institution. To reduce confounding, NOM patients were frequency-matched to OM patients by age (±5 years), sex, and mechanism of injury, yielding 39 OM and 44 NOM patients.

FAST examinations were performed per ATLS^®^ protocol by the first-contact physician and confirmed by the trauma surgeon; CT scans were subsequently obtained except in patients requiring immediate laparotomy. NOM consisted of close clinical and laboratory monitoring, with failure prompting surgery. Collected admission variables included lactate, heart rate, leukocyte count, FAST result, mechanism of injury, AAST grade, and ISS.

Continuous variables were presented as medians (IQR) and compared using Mann–Whitney U tests, while categorical variables were analyzed with chi-square or Fisher’s exact tests. The diagnostic accuracy of lactate was evaluated using ROC curves, with Optimal thresholds derived from AUC analysis and the Youden index; sensitivity, specificity, LR^+^, and LR^−^ for the clinically relevant cut-off of 3.5 mmol/L were calculated with 95% CIs.

A multivariable logistic regression model incorporating lactate, heart rate, leukocytes, and FAST positivity chosen for their clinical relevance and immediate availability was developed to estimate the probability of OM.

The final model equation was as follows: logit(p) = −11.238565 + 0.591184 (lactate) + 0.037517 (heart rate) − 0.114149 (leukocytes) + 7.362468 (FAST-positive).

Internal validation was performed using 1000 bootstrap resamples, computing optimism-corrected AUC and Brier scores following TRIPOD recommendations. Analyses were performed using Python 3.10 (NumPy, SciPy, scikit-learn, Wilmington, DE, USA) for model development and validation, SPSS v26 (Chicago, IL, USA) was used for descriptive statistics, and GraphPad Prism v9 (San Diego, CA, USA) was used for visualizations.

## 3. Results

In total, 83 trauma patients were included, all of whom were assessed upon admission to the emergency department. The median age was 35 years (IQR 23–45 years), and 70 of the patients were male (84.3%). Regarding clinical outcomes, 39 patients (47.0%) required OM, while 44 patients (53.0%) had NOM with success rate of 100%. Mechanisms of injury were diverse: motorcycle incidents in 26.5% (*n* = 22), car incidents in 22.9% (*n* = 19), run-over incidents in 19.3% (*n* = 16), assaults in 15.7% (*n* = 13), falls from height in 9.6% (*n* = 8), and other unspecified mechanisms in 6.0%, (*n* = 5). FAST revealed abdominal fluid in 64 patients (77.1%). These baseline data are summarized in Table 1.

**Figure 1:**
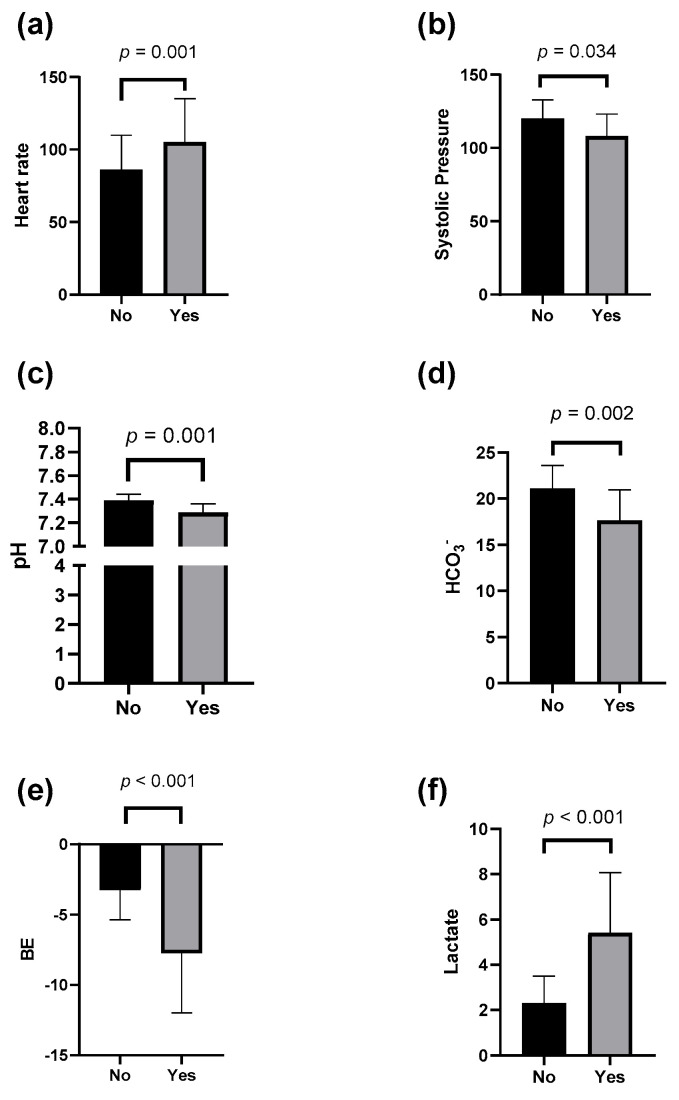
Comparison of clinical and laboratory variables between patients who underwent OM and NOM using Mann–Whitney U test.

Comparative analysis with a Mann–Whitney U test of physiological and biochemical parameters at emergency department admission revealed significant differences between patients who underwent OM and those selected for NOM. Patients requiring OM exhibited significantly higher heart rates (HR) (Figure 1a; *p* = 0.001) and slightly lower SBP (Figure 1b; *p* = 0.034); arterial blood pH was significantly lower in patients who underwent OM (Figure 1c; *p* = 0.001), paralleled by a significant reduction in serum HCO_3_^−^ levels (Figure 1d; *p* = 0.002) and BE (Figure 1e; *p* < 0.001). Finally, and most notably, serum lactate concentrations were significantly elevated in patients who underwent OM (Figure 1f; *p* < 0.001).

**Figure 2:**
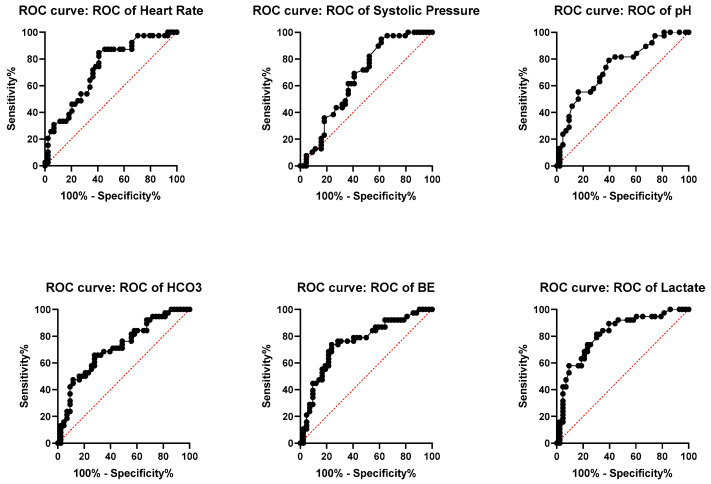
Discriminatory performance of quantitative variables to predict requirement of OM. Heart Rate: AUC: 7264; 95% confidence Interval: 0.6179 to 0.8349, *p* = 0.0002. Systolic Preassure: AUC: 0.6632; 95% confidence Interval: 0.5457 to 0.7806, *p* = 0.0106. Ph: AUC: 0.7390; 95% confidence Interval: 0.6311 to 0.8469, *p* = 0.0002. HCO_3_^−^: AUC: 0.7170; 95% confidence Interval: 0.6053 to 0.8286, *p* = 0.0008. BE: AUC: 0.7544; 95% confidence Interval: 0.6460 to 0.8627, *p* < 0.0001. Lactate: AUC: 0.8152; 95% confidence Interval: 0.7209 to 0.9095, *p* < 0.0001.

To evaluate the predictive accuracy of variables with significant differences between patients who underwent OM and NOM, AUROC analyses were performed and 95% CI and *p*-values were calculated for each one of the variables in order to determine their individual discriminatory capacity to identify patients who require OM. Results are depicted in Figure 2.

Serum lactate levels demonstrated the highest predictive accuracy among all variables with an AUROC of 0.8152 (95% CI: 0.7209–0.9095, *p* < 0.0001), indicating strong discriminative power to identify patients needing OM. This was closely followed by BE, with an AUROC of 0.7544 (95% CI: 0.6460–0.8627, *p* < 0.0001), arterial pH, with an AUROC of 0.7390 (95% CI: 0.6311–0.8469, *p* = 0.0002), and serum HCO_3_^−^, with an AUROC of 0.7170 (95% CI: 0.6053–0.8286, *p* = 0.0008). Heart rate had a moderate predictive value, with an AUROC of 0.7264 (95% CI: 0.6179–0.8349, *p* = 0.0002), while systolic blood pressure had the lowest AUROC among the variables, with 0.6632 (95% CI: 0.5457–0.7806, *p* = 0.0106), suggesting limited predictive capacity as a standalone parameter.

Given its strong discriminatory capacity, admission lactate was examined at the clinically meaningful threshold of 3.5 mmol/L. At the clinically relevant threshold of 3.5 mmol/L, lactate showed a sensitivity of 0.737 (95% CI 0.569–0.866), a specificity of 0.744 (95% CI 0.588–0.865), an LR^+^ of 2.88 (95% CI 1.67–4.96), and an LR^−^ of 0.35 (95% CI 0.20–0.62) as detailed in Supplementary Table S1. A multivariable logistic regression model was constructed using the four admission variables available during the early ED evaluation—lactate, heart rate, leukocyte count, and FAST result—which produced the following final equation: logit(p) = −11.238565 + 0.591184 (lactate) + 0.037517 (HR) − 0.114149 (WBC) + 7.362468 (FAST-positive) (Table S2), achieving an apparent AUC of 0.904 and a Brier score of 0.126. Bootstrap internal validation using 1000 resamples demonstrated a mean optimism of 0.022, producing an optimism-corrected AUC of 0.882 and a corrected Brier score of 0.149, indicating good model performance for an exploratory early-triage tool (Table S3). Institutional biochemical and hematological reference intervals used for laboratory interpretation of patient results are provided in Table S4.

## 4. Discussion

In this study, we examined admission physiological, biochemical, and sonographic parameters as early predictors of the need for operative management in adult patients with blunt abdominal trauma. Our findings show that serum lactate, FAST positivity, heart rate, and leukocyte count, all variables obtainable within minutes of arrival, carry meaningful discriminatory value for identifying patients at higher risk of requiring early surgical intervention. Among these, lactate demonstrated the strongest individual performance, consistent with its well-established role as a surrogate for tissue hypoperfusion and occult shock. The observed AUC of 0.815, along with balanced sensitivity and specificity at the clinically relevant threshold of 3.5 mmol/L, supports lactate’s utility as an initial triage marker, even when traditional indicators such as systolic blood pressure remain within normal limits.

Trauma is a worldwide public health problem. Its global burden on society is explained by the fact that it is the cause of more than 10% of deaths and disabilities in people of working age. According to the reviewed articles, men are most affected by injuries (56.2–94.2%), with a mean age between 23.9 and 56.5 years [37,38,39,40,41,42]. In our study, we also found a predominance in male patients (84.3%) with mean age of 35 years; these numbers are within the ranges reported in the international literature; slight discrepancies could be explained by demographic differences between the countries where the studies were conducted.

All over the world, traffic incidents are the leading cause of blunt trauma (22.5–90%), involving motorcycles (34.9–68.4%), cars and other similar vehicles (10.9–65%), pedestrians (11.6%), and bicycles (6.2%); these are closely followed by assaults (18–31.2%), falls on flat ground and other heights (10–22.4%), incidents with horses (3.1–10.5%), and falling objects (3.5%) [43]. According to the articles consulted, the distribution of mechanisms of injury has a similar trend in pediatric patients with an increase in physical aggression (31.2%), associated with a relative decrease in traffic incidents (34.3%) [40]. It is worth mentioning that international information includes a considerable number of unspecified causes (5.1–82.1%), which may be due to grouping several causes with lower frequencies into a single category or the lack of such data in clinical records. In our population, mechanisms of injury had a similar distribution to that observed in the revised studies, including 6% of unspecified causes.

From the description of the Pringle maneuver in 1908 until the 1970s, OM was the gold standard for the management of solid organ injuries due to blunt trauma [17], and a positive FAST, the report of an AAST grade III, or greater solid organ injury was sufficient to admit a patient to the operating room; however, the large number of reports of mortality, morbidity, and the economic burden of NOM vs. OM (including non-therapeutic laparotomies) have made the latter the current standard of care for all adult patients with solid organ injuries due to blunt trauma and without peritoneal signs or irreversible hemodynamic instability, regardless of the magnitude of anatomical damage determined by the AAST injury scoring scale, with success rates over 80% [44]. Anca et al. even observed that the benefits of NOM could be extended to the pediatric population with similar clinical behavior [40]. In the reviewed literature, NOM was offered in 46.5–93.3% of patients with success rates between 74 and 100% [45]. In our study, 53% of patients underwent NOM with a 100% success rate, which corresponds to international numbers.

In our analysis, the Focused Assessment with Sonography for Trauma (FAST) emerged as the predictor with the highest discriminative value for operative management (OM) versus non-operative management (NOM), even though it was penalized in the Firth logistic regression model due to partial separation. This statistical adjustment was necessary to reduce bias from the small sample size and the quasi-complete separation introduced by FAST, yet the magnitude of its contribution to the model underscores its clinical importance. In practical terms, positive FAST findings substantially increased the probability of surgical intervention, supporting its role as a cornerstone in the early evaluation of blunt abdominal trauma. Nevertheless, it is important to recognize the limitations of FAST as a diagnostic modality. While its reported sensitivity (78.9%) and specificity (99.2%) are high, FAST remains a dichotomous screening test and cannot quantify intraperitoneal fluid volume, differentiate fluid characteristics, grade organ injuries, or assess retroperitoneal structures. Consequently, a negative FAST does not definitively rule out significant intra-abdominal bleeding. This reinforces the principle that FAST findings should always be interpreted in conjunction with clinical context and adjunctive parameters. Another key consideration is that hemodynamic instability does not always manifest as hypotension or tachycardia. Shock may be present in the absence of overt vital sign abnormalities, and conversely, abnormal vital signs may not always indicate true hypoperfusion. Alterations in heart rate and blood pressure often represent late decompensation, when compensatory mechanisms are exhausted, and are strongly associated with increased morbidity and mortality even under timely intervention. Therefore, reliance solely on physical examination or traditional vital signs may delay the recognition of evolving shock states. In this setting, biochemical markers such as lactate, pH, and base excess become essential complements to FAST. Elevated lactate, decreased pH, and reduced base excess serve as early indicators of occult hypoperfusion and are strongly correlated with surgical need in our cohort. Integrating these metabolic parameters with FAST findings provides a more comprehensive assessment of the physiological reserve and injury burden, thereby improving the accuracy of surgical decision-making. Taken together, our results suggest that, while the statistical penalization of FAST in Firth regression reflects the methodological challenges of small-sample modeling, the clinical weight of a positive FAST remains considerable. Its predictive value is further enhanced when combined with physiological and biochemical parameters, reinforcing the need for multimodal assessment in trauma care [31,32,44,46].

Hemorrhage is responsible for 30–50% of deaths in trauma patients within the first 48 h after hospital admission, increases by 1% for every 3 min of delay in definitive treatment, and is the leading cause of preventable death in those cases (16%), due to massive exsanguination and multiple organ failure resulting from prolonged shock. Solid organ injuries are a frequent cause of bleeding in trauma patients. For this reason, clinical and laboratory signs suggesting considerable blood loss are often considered indicators of OM requirement in this population; however, there is little information on cutoff values that could support such decisions.

The model demonstrated excellent apparent discrimination (AUC 0.904) and maintained strong performance after internal bootstrap validation (optimism-corrected AUC 0.882), suggesting reasonable stability for an exploratory analysis despite the modest sample size. These findings indicate that integrating lactate with FAST and basic physiological parameters may offer meaningful early insight into which patients are more likely to require operative intervention. This may be particularly relevant in settings where CT availability is limited or delayed, where early-triage decisions are most challenging, and where the rapid identification of high-risk patients could facilitate expedited operative readiness or early mobilization of surgical resources. Nevertheless, this study does not propose a definitive decision rule. Instead, it provides an initial, transparent, and reproducible framework that emphasizes early, admission-based variables and internal validation. Given the single-center nature of our cohort and the limited number of operative events, the proposed model should be interpreted cautiously and not applied in isolation to guide clinical management. Trauma care is multifactorial, and operative decisions should continue to rely on comprehensive clinical assessment, evolving hemodynamic status, and definitive imaging when available. Our results should therefore be viewed as hypothesis-generating and as a foundation for more robust validation and refinement in future multicenter studies.

## 5. Limitations

This study has important limitations. First, it was conducted at a single public trauma center with a relatively small sample size (83 patients and 39 surgical events), which increases the risk of overfitting, despite the use of internal bootstrap validation. The number of events per variable is modest for a four-predictor logistic regression model, and therefore the results should be interpreted as exploratory rather than definitive. Second, although the model uses only physiological, biochemical, and FAST findings available during the first minutes of evaluation, clinical and operational differences across institutions—such as variability in trauma team structure, resuscitation practices, or access to imaging—may limit its direct generalizability. Third, the study did not include patients treated with laparoscopy or endovascular therapy, as these modalities were not available at our institution, and therefore the model applies only to settings where early management relies primarily on FAST-guided assessment and laparotomy. Fourth, while internal bootstrap validation demonstrated good optimism-corrected discrimination and acceptable calibration, the model has not yet undergone external validation in independent cohorts. Finally, the model is intended to support, “not replace”, clinical judgment, trauma guidelines, or definitive imaging, and should not be used as a standalone criterion for surgical decision-making.

## 6. Future Perspectives

Future work should focus on validating this early-triage model in larger, multicenter cohorts that include diverse trauma populations, varied injury mechanisms, and broader access to advanced interventions such as embolization or damage-control laparoscopy. Prospective studies are warranted to evaluate how integrating early biochemical and sonographic parameters into structured triage algorithms may improve time-to-decision and reduce delays to operative intervention in hemodynamically tenuous patients. In addition, exploring dynamic modeling approaches that incorporate serial lactate measurements, trends in vital signs, or machine-learning techniques may allow for improved prediction accuracy while preserving interpretability. Ultimately, external validation and prospective implementation studies are essential before considering clinical integration, and future efforts should ensure that any triage tool maintains transparency, reproducibility, and patient safety as foundational principles.

## 7. Conclusions

In this exploratory study, admission lactate, FAST positivity, heart rate, and leukocyte count demonstrated meaningful early discriminatory value for identifying blunt trauma patients who may require operative management. Admission lactate showed the strongest individual performance, with a clinically relevant threshold of 3.5 mmol/L, providing balanced sensitivity and specificity. The four-variable logistic regression model developed from these admission-only parameters demonstrated excellent apparent discrimination and maintained strong performance after internal bootstrap correction, supporting its potential utility as an early-triage aid before CT imaging is available. However, given the single-center design, modest sample size, and absence of external validation, the model should be interpreted cautiously and not used to guide operative decisions in isolation. Rather, these findings serve as a foundation for future multicenter studies aimed at developing reliable, reproducible, and clinically safe early-triage tools in blunt abdominal trauma.

## Figures and Tables

**Table 1:** Baseline demographic and clinical characteristics of trauma patients.

	Patients (*n* = 83)
Age in years, median (IQR)	35 (23–45)
Male sex, *n* (%)	70 (84.3)
Operative management, *n* (%)	
Yes	39 (47)
No	44 (53)
Mechanisms of injury, *n* (%)	
Motorcycle incidents	22 (26.5)
Assaults	13 (15.7)
Car incidents	19 (22.9)
Run-over incidents	16 (19.3)
Falls from height	8 (9.6)
Others	5 (6)
FAST (+)	64 (77.1)

## Data Availability

Raw data are available directly from the corresponding authors with a reasonable request.

## Supplementary Materials

The following supporting information can be downloaded at https://www.mdpi.com/article/10.3390/jcm14238379/s1, Table S1: Diagnostic Accuracy of Lactate ≥ 3.5 mmol/L; Diagnostic accuracy metrics for lactate ≥ 3.5 mmol/L; Table S2: Final Multivariable Logistic Regression Model; Table S3: Bootstrap Internal Validation Results (1000 Resamples); Table S4: Institutional Reference Intervals for Biochemical and Hematological Parameters (IMSS-BIENESTAR, Puebla).
