# Supplementary material for: Development and Internal Validation of a Predictive Model for Operative Management in Blunt Abdominal Trauma Using Admission Physiological and Biochemical Parameters
Source: J Clin Med. 2025 Nov 26;14(23):8379. doi: 10.3390/jcm14238379 (PMC12693253; doi:10.3390/jcm14238379)
Supplement: Supplementary file 1 [file jcm-14-08379-s001.zip › jcm-3939433-supplementary.pdf]

Supplementary Materials

Supplementary Table S1. **Diagnostic accuracy of lactate ≥ 3.5 mmol/L, including the 2×2 contingency table and detailed estimates of sensitivity, specificity, positive likelihood ratio (LR^+), and negative likelihood ratio (LR^-).**

Lactate Category	OM = 1	OM = 0
≥ 3.5 mmol/L	28	11
< 3.5 mmol/L	10	32

Diagnostic accuracy metrics for lactate ≥ 3.5 mmol/L:

Parameter	Estimate (95% CI)
Sensitivity	0.737 (0.569–0.866)
Specificity	0.744 (0.588–0.865)
Positive likelihood ratio (LR+)	2.88 (1.67–4.96)
Negative likelihood ratio (LR–)	0.35 (0.20–0.62)

Supplementary Table S2. Final multivariable logistic regression model presenting the full equation and regression coefficients for lactate, heart rate, leukocyte count, and FAST status

Final model equation:

logit(p) = -11.238565 + 0.591184(lactate) + 0.037517(HR) - 0.114149(WBC) + 7.362468(FAST-positive) FAST-positive = 1 if FAST is positive, 0 otherwise.

Variable	Coefficient (β)
Intercept	−11.238565
Lactate (mmol/L)	0.591184
Heart rate (bpm)	0.037517
Leukocytes ($\times 10^9$/L)	−0.114149
FAST positive	7.362468

Supplementary Table S3. Bootstrap internal validation results (1,000 resamples), including apparent AUC, optimism-corrected AUC, and Brier scores.

Performance Metric	Apparent Value	Mean Optimism	Corrected Value
AUC	0.904	0.022	0.882
Brier score	0.126	−0.023	0.149

Supplementary Table S4. I Institutional reference intervals for biochemical and hematological parameters used in this study (IMSS-BIENESTAR, Puebla). Reference values correspond to institutional ranges provided by the IMSS-BIENESTAR Clinical Laboratory (Hospital de Traumatología y Ortopedia “Dr. Rafael Moreno Valle”, Puebla, Mexico). These intervals were used for interpretation of all patient results in this study.

**Parameter**	**Unit**	**Reference Interval**
Gasometry (Venous, 37°C)		
pH	–	7.35 – 7.45
pCO_2	mmHg	35 – 45
pO_2	mmHg	80 – 100
HCO_3^-	mmol/L	22.0 – 28.0
Base Excess	mmol/L	–2.0 – +3.0
SO_2	%	60 – 80 (venous)
Electrolytes		
Sodium (Na^+)	mmol/L	135 – 145
Potassium (K^+)	mmol/L	3.5 – 5.0
Chloride (Cl^-)	mmol/L	98 – 106
Calcium (Ca^{2+}, total)	mg/dL	8.5 – 10.5
Magnesium (Mg^{2+})	mg/dL	1.6 – 2.3
Phosphate (PO_4^{3-})	mg/dL	2.5 – 4.5
Renal Function		
Urea	mg/dL	15 – 40
Creatinine (men)	mg/dL	0.66 – 1.25
Liver Function		
Total Bilirubin	mg/dL	0.2 – 1.0
Direct Bilirubin	mg/dL	0.0 – 0.3
Indirect Bilirubin	mg/dL	0.2 – 0.7
ALT (Alanine aminotransferase)	U/L	Men: <41
AST (Aspartate aminotransferase)	U/L	Men: 17 – 59
Alkaline Phosphatase	U/L	40 – 130
GGT (Gamma-glutamyltransferase)	U/L	Men: 15 – 73
LDH (Lactate dehydrogenase)	U/L	140 – 246
Other Biochemistry		
Glucose	mg/dL	74 – 106
Lactate	mmol/L	0.5 – 2.2
Procalcitonin	ng/mL	<0.5 low risk sepsis; >2 high risk
Albumin	g/dL	3.5 – 5.0
Globulin	g/dL	2.7 – 3.8
A/G Ratio	–	1.0 – 2.1
Hematology		
Leukocytes	$\times 10^3/\mu L$	4.0 – 12.0

Neutrophils (%)	%	40 – 74
Lymphocytes (%)	%	19 – 48
Hemoglobin (men)	g/dL	13.0 – 18.0
Hematocrit (men)	%	40 – 54
Platelets	$\times 10^3/\mu L$	150 – 450
